# Stimulation of TRPV1+ peripheral somatosensory nerves suppress inflammation via the somato-autonomic reflex

**DOI:** 10.1016/j.isci.2025.111831

**Published:** 2025-01-17

**Authors:** Dengcen Song, Zheng Cao, Yong Hu, Fengyu Mao, Cheng Cao, Zijing Liu

**Affiliations:** 1 Beijing Institute of Biotechnology, Beijing, China; 2 School of Life and Health Sciences, Hubei University of Technology, Wuhan, China

**Keywords:** Neuroscience, Sensory neuroscience

## Abstract

Excessive inflammation causes a wide range of diseases. Here, we found that stimulating TRPV1+ nerves at the nape activated the nucleus of the solitary tract and C1 neurons in the brainstem via the somatosensory afferent pathway, and rapidly induced the secretion of corticosterone, and drove the vagal-adrenal axis to release serum catecholamines, and activated the autonomic-splenic reflex to suppress cytokine production. RNA sequencing (RNA-seq) analysis revealed that stimulating TRPV1+ nerves significantly changed the expression of genes enriched in multiple pathways related to the inflammatory response in the spleen under pathological and normal physiological conditions. TRPV1 agonist lost these anti-inflammatory effects in *trpv1ko* mice. Our study revealed a neural circuit that stimulating TRPV1+ somatosensory afferents at the nape could concurrently drive the sympathetic and parasympathetic efferents to synergistically induce anti-inflammatory effects. Furthermore, stimulation of TRPV1+ peripheral sensory afferents in specific body regions is an efficient therapeutic approach to treat inflammatory diseases.

## Highlights

• Stimulation of TRPV1+ peripheral nerve attenuates systemic inflammation
• Somato-autonomic reflex induces the secretion of catecholamines
• Activating TRPV1+ afferent at nape drives sympathetic and vagal efferent pathways
• TRPV1+ somatosensory afferent stimulating modulates the expression of splenic genes

## Graphical abstract

**Figure:**
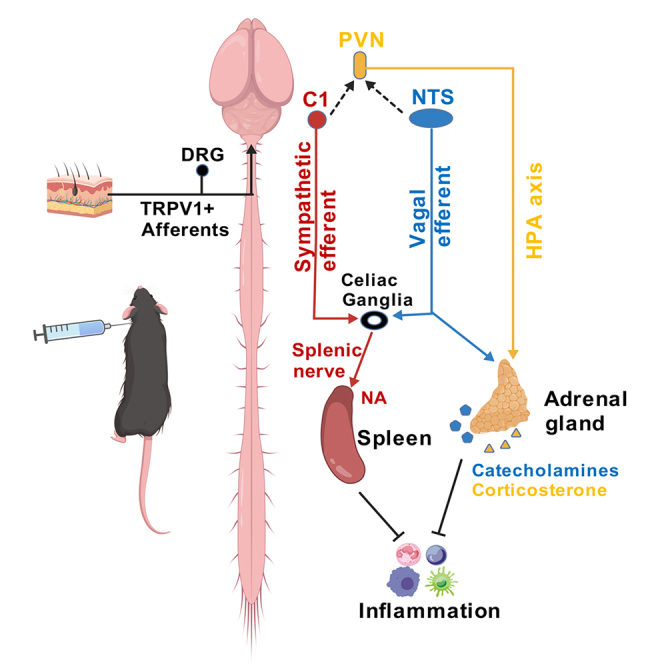

## Introduction

Inflammation can eliminate invading pathogens and restore physiological homeostasis, but an excessive or chronic inflammatory response damages various tissues and induces multiple diseases. To date, effective prevention or treatment methods are lacking. Moxibustion and apitherapy, which can produce anti-inflammatory responses and analgesia and treat a wide range of diseases, have long been used in traditional oriental medicine, but their therapeutic mechanism remains unclear. Moxibustion-like stimulation (>43°C) can activate peptidergic C-fibers in the skin.^1^ Ginger-partitioned moxibustion or garlic-partitioned moxibustion, in which gingerol and allicin are agonists of TRPV1, can increase the therapeutic effects. Melittin, the main peptide in bee venom, activates TRPV1 ion channels and TRPV1+ peripheral sensory nerves.^2^ Therefore, we speculate that stimulation of TRPV1+ peripheral afferents may modulate the inflammatory response.

TRPV1, a nonselective cation channel, is a thermoreceptor activated by noxious heat (>43°C) and some endogenous and exogenous mediators.^3^^,^^4^ TRPV1 is highly expressed in dorsal root ganglia (DRG) and nodose ganglion (NG), which transmit somatosensory and vagal sensory information, respectively. Based on single-cell RNA sequencing (RNA-seq) and function assay, the sensory neurons of DRG are divided into 11 distinct cell types. TRPV1 is selectively expressed in NP2, NP3, and PEP1 subgroups of nociceptors.^5^^,^^6^^,^^7^ Our previous studies indicated that the percentage of TRPV1+ nociceptors in total DRG neurons is approximately 20%, and TRPV1+ primary afferents project to lamina I and outer layer of lamina II within the dorsal horn of the spinal cord.^8^ Noxious and/or thermal stimuli from the skin and deep tissues are further transmitted to several brain regions. NG contains 18 types of vagal neurons. TRPV1 is specifically expressed in NG12–NG16 neuron subtypes that transmit harmful or inflammatory signaling of internal organs to the nucleus of the solitary tract (NTS) in the brainstem.^9^ Then, the autonomic efferent nerves are activated to modulate visceral functions.

Accumulating evidence indicates that electric stimulation of nerves can regulate peripheral immune responses. Electric stimulation of vagal afferent and efferent nerves can attenuate systemic inflammation.^10^^,^^11^ Electroacupuncture at specific somatic tissues activates autonomic nervous system to induce either anti- or pro-inflammatory effects.^12^^,^^13^^,^^14^ Here, we revealed that thermal or chemical stimulation of TRPV1+ peripheral somatosensory nerves at nape could suppress inflammatory response via somato-autonomic reflex.

## Results

### TRPV1+ peripheral nerve stimulation induced the anti-inflammatory effects

**Figure 1:**
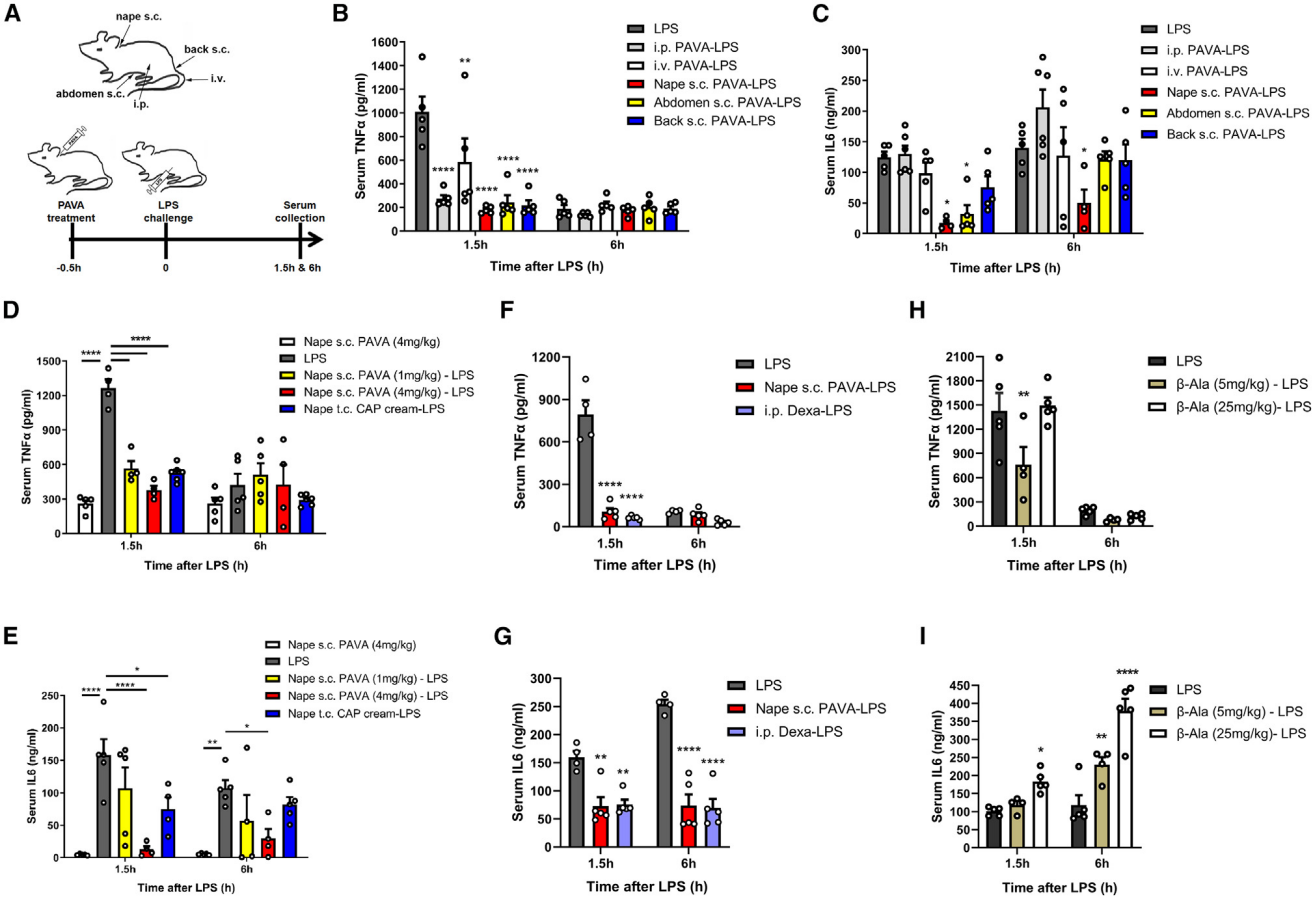
Stimulation of peripheral sensory afferents modulated inflammation (A) Schematic of distinct PAVA treatments and timeline of the experiments. (B and C) PAVA treatments in different body areas inhibited TNF-α (B) and IL-6 (C). (*n* = 4–6/group). (D and E) Distinct PAVA treatments at nape affected TNF-α (D) and IL-6 (E). (*n* = 4–6/group). (F and G) PAVA or dexamethasone treatment suppressed the release of TNF-α (F) and IL-6 (G). (*n* = 4–5/group). (H and I) β-alanine nape s.c. affected serum level of TNF-α (H) and IL-6 (I). (*n* = 4–5/group). Data are presented as the mean ± SEM. Two-way ANOVA with Bonferroni post tests, ∗∗∗∗*p* < 0.0001, ∗∗∗*p* < 0.001, ∗∗*p* < 0.01, ∗*p* < 0.05.

Nonivamide or pelargonic acid vanillylamide (PAVA), a less-pungent capsaicin analog, is a specific agonist of TRPV1 ion channel and used for anti-inflammation and relief of muscle pain in clinical application.^15^^,^^16^ The toxicity of PAVA was evaluated by different administration routes. In our experiments, PAVA was administered at a dose of 4 mg/kg intraperitoneally (i.p.), 4 mg/kg subcutaneously (s.c.) into the nape (hereafter PAVA nape s.c.), 4 mg/kg s.c. into the abdomen (hereafter PAVA abdomen s.c.), 4 mg/kg s.c. into the back close to the tail (hereafter PAVA back s.c.), or 0.25 mg/kg intravenously (i.v.) into the tail veins (Figure 1A). No signs of toxicity or death were found in the PAVA-treated mice. Previous studies have indicated that electroacupuncture stimulation at specific body regions (acupoints) can activate distinct neural circuits and modulate systemic inflammation in a somatotopy-dependent manner (body region specificity), while stimulation at non-acupoints fails to induce anti-inflammatory effects.^12^^,^^13^^,^^14^ Therefore, we designed a non-overlap layout scheme to select distinct injection positions on the body. PAVA was administrated via different routes to assess whether stimulating TRPV1+ afferents at specific body regions can modulate systemic inflammation induced by lipopolysaccharide (LPS), a bacterial endotoxin, through the specific neural pathway. LPS injected i.p. significantly increased serum levels of proinflammatory cytokines, including tumor necrosis factor alpha (TNF-α), interleukin (IL)-6, and IL-1β. Different PAVA treatments were performed 30 min before LPS challenge. Our PAVA treatments all substantially inhibited TNF-α at 1.5 h following LPS injection (Figure 1B), when the release of TNF-α largely peaked in the serum. Notably, PAVA nape s.c. substantially reduced IL-6 at both 1.5 h and 6 h after LPS administration, whereas PAVA abdomen s.c. inhibited only IL-6 at 1.5 h, and the other treatments did not significantly reduce IL-6 (Figure 1C). Serum IL-1β was barely detected at 1.5 h. Both PAVA nape s.c. and PAVA back s.c. caused a reduction in IL-1β at 6 h (Figure S1A). Based on these results, PAVA nape s.c. was the most efficient anti-inflammatory method among the indicated treatments, suggesting that stimulation of TRPV1+ peripheral sensory afferents can inhibit cytokine production. PAVA nape s.c. was chosen for subsequent studies.

Without LPS challenge, PAVA nape s.c. evoked baseline expression of TNF-α and IL-6 in serum (Figures 1D and 1E). The anti-inflammatory effect of PAVA treatment was dose dependent. TNF-α level was reduced by 55.3% with 1 mg/kg PAVA and by 70.1% with 4 mg/kg PAVA (Figure 1D). IL-6 was moderately but not significantly decreased with 1 mg/kg PAVA, while IL-6 was strongly reduced by 92% at 1.5 h and by 72.4% at 6 h with 4 mg/kg PAVA (Figure 1E). Furthermore, CAP cream (PAVA 0.75 mg/g + capsaicin 0.25 mg/g) was transcutaneously (t.c.) applied to the shaved nape, which substantially reduced TNF-α and IL-6 (Figures 1D and 1E), suggesting that stimulation of TRPV1+ cutaneous sensory afferents directly induce anti-inflammatory effects. Glucocorticoids are the most potent class of anti-inflammatory drugs and clinically used for treating various inflammatory diseases, but the severe side effects of glucocorticoid-treatments limit their long-term application, such as osteoporosis and insulin resistance. Dexamethasone, the synthetic glucocorticoid receptor agonist, was i.p. injected into the mice before LPS administration. PAVA nape s.c. inhibited the LPS-evoked release of TNF-α and IL-6 similar to dexamethasone pretreatment (Figures 1F and 1G), suggesting a therapeutic potential of TRPV1+ peripheral afferent activation for treating inflammatory diseases. MRGPRD+ nociceptors are responsible for mechanical stimuli and are selectively activated by β-alanine to induce itch, and they constitute approximately 30% of total DRG neurons.^8^^,^^17^^,^^18^^,^^19^ Single-cell RNA-seq studies indicated that MRGPRD was selectively expressed in NP1 subgroup and that TRPV1 was expressed in NP2, NP3, and PEP1 clusters in DRG.^5^^,^^6^^,^^7^ Pretreatment with β-alanine nape s.c. suppressed the LPS-evoked release of TNF-α but it increased IL-6 expression (Figures 1H and 1I), indicating that stimulation of MRGPRD+ afferents induces a complex inflammatory response.

**Figure 2:**
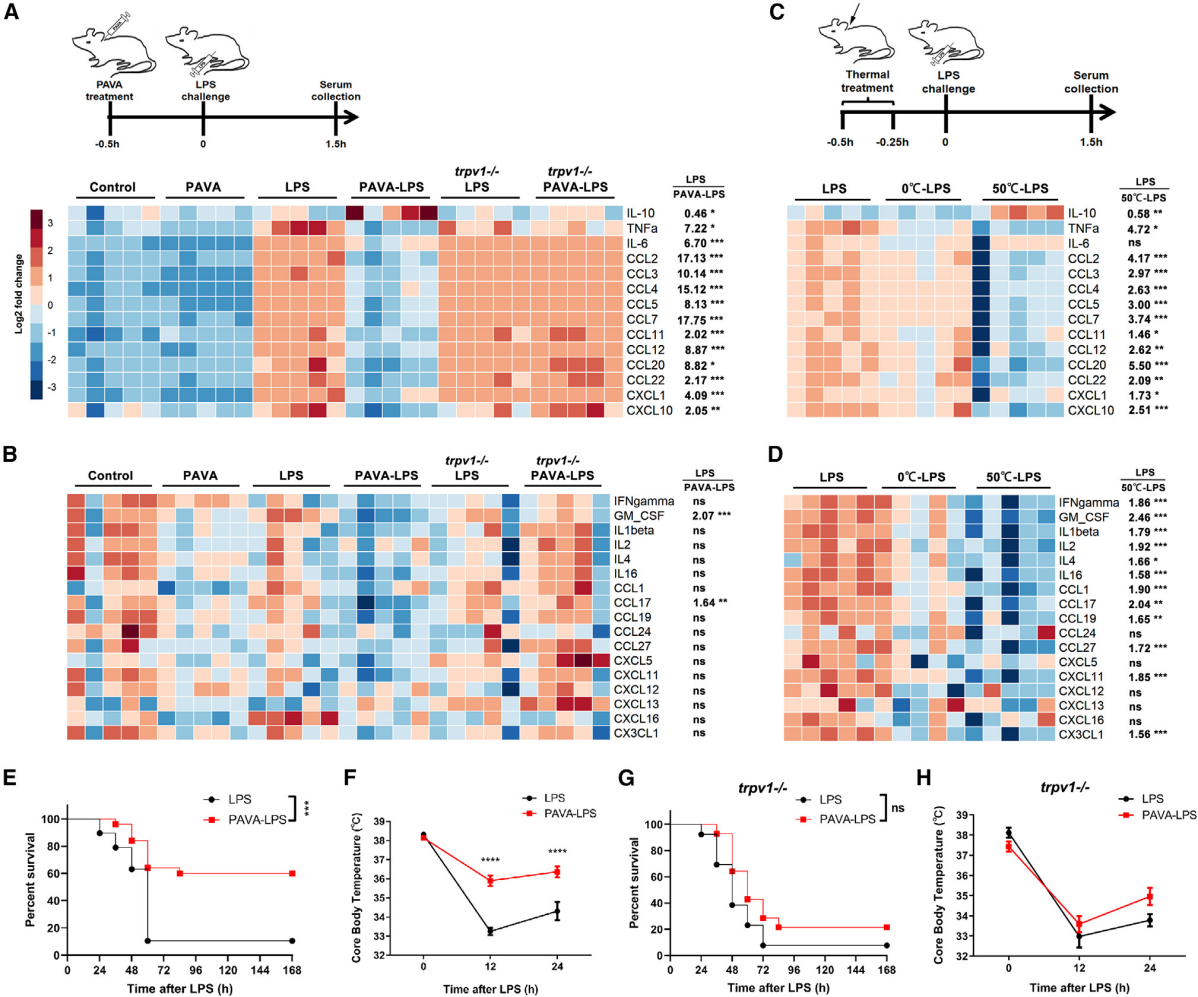
TRPV1+ peripheral somatosensory nerve stimulating protected the host against severe systemic inflammation (A and B) Heatmap showing serum cytokine levels at 1.5 h after distinct treatments. Fold change between LPS-only group and PAVA-LPS group (right). (*n* = 5/group). (C and D) With heat stimulation (∼50°C) or cold stimulation (∼0°C) of the shaved nape, heatmap showing serum cytokine levels at 1.5 h following LPS challenge. Fold change between LPS-only group and 50°C-LPS group (right). (*n* = 5/group). (E) PAVA nape s.c. increased the survival rate of endotoxemic mice (*n* = 25) compared to untreated endotoxemic littermates (*n* = 19). (F) PAVA nape s.c. improved the core body temperature of endotoxemic mice (*n* = 25) compared to untreated endotoxemic littermates (*n* = 19). (G) PAVA nape s.c. could not increase the survival rate of *trpv1ko* endotoxemic mice (*n* = 14) compared to untreated endotoxemic littermates (*n* = 13). (H) PAVA nape s.c. could not improve the core body temperature of *trpv1ko* endotoxemic mice (*n* = 17) compared to untreated endotoxemic littermates (*n* = 12). Data are presented as the mean ± SEM. Unpaired two-sided Student’s t test (A–D), Mantel-Cox log rank test (E and G), or two-way ANOVA with Bonferroni post tests (F and H). ∗∗∗∗*p* < 0.0001, ∗∗∗*p* < 0.001, ∗∗*p* < 0.01, ∗*p* < 0.05; ns, not significant.

Severe systemic inflammation is induced by the excessive release of a series of proinflammatory cytokines, and successful treatment is required to inhibit most of these cytokines rather than only a single cytokine. We examined the serum levels of 31 cytokines through a Luminex assay. Without LPS challenge, PAVA treatment significantly decreased the basal level of IL-6 and did not affect the expression of other cytokines (Figures 2A and 2B). Luminex analysis revealed that LPS injection substantially elevated serum levels of 13 cytokines (fold > 1.5, *p* < 0.05) at 1.5 h, including TNF-α, IL-6, CCL2, CCL3, CCL4, CCL5, CCL7, CCL11, CCL12, CCL20, CCL22, CXCL1, and CXCL10. All of them were significantly inhibited by PAVA pretreatment, whereas the anti-inflammatory cytokine IL-10 was increased (Figure 2A).

Capsaicin, a specific agonist of TRPV1 ion channel, can suppress inflammation via a TRPV1-independent mechanism by directly targeting PKM2-LDHA and COX2 in sepsis.^20^ To confirm whether TRPV1 ion channel is the actual target of PAVA in modulating its anti-inflammatory effect, we compared cytokine expression between wild-type and *trpv1ko* endotoxemic mice. Luminex analysis showed that the anti-inflammatory effect of PAVA treatment was completely reversed in *trpv1ko* mice (Figures 2A and 2B). Furthermore, PAVA administration did not inhibit IL-1β expression in *trpv1ko* endotoxemic mice at 6 h following LPS challenge (Figure S1B). Thus, PAVA treatment induced anti-inflammatory effects via TRPV1 ion channel. To investigate the contribution of TRPV1+ neurons and nerves in PAVA-induced anti-inflammatory response, mice were systemically treated with resiniferatoxin (RTX) to specifically induce TRPV1+ sensory neuropathy. Our previous studies indicated that TRPV1+ neurons in DRG can be divided into TRPV1^high^ and TRPV1^low^ subtypes according to the relatively high or low expression level of TRPV1.^8^^,^^21^ The number of TRPV1^high^ neurons in DRG was significantly reduced in RTX-treated mice compared to control mice, while RTX treatment did not affect TRPV1^low^ neurons (Figures S2A and S2B). TRPV1+ neuron ablation impaired the response to noxious heat (Figure S2C). RTX-mediated sensory denervation significantly suppressed PAVA-induced anti-inflammatory effects on reducing the expression of TNF-α and IL-6 (Figures S2D and S2E). Taken together, PAVA treatment induced anti-inflammatory effects through TRPV1+ sensory neurons and afferents.

Thermal stimulation (>43°C) can elicit discharges of TRPV1+ cutaneous afferents. Heat stimulation (∼50°C) or cold stimulation (∼0°C) on the shaved nape was applied for 15 min (Figure 2C). Luminex analysis showed that more cytokines were inhibited by heat stimulation than by PAVA treatment, while the administration of 4 mg/kg PAVA more effectively reduced the levels of some cytokines, such as IL-6 and CCL2, than heat treatment (Figures 2A–2D). Moreover, heat stimulation promoted IL-10 expression in a manner similar to PAVA treatment. In contrast, only TNF-α and CCL3 levels were significantly decreased by cold stimulation (Figures 2C and 2D). Therefore, stimulation of TRPV1+ cutaneous afferents at the nape with moxibustion-like treatment effectively suppressed proinflammatory cytokine production.

Among the LPS-challenged mice, 60% of the mice pretreated with PAVA survived, while only 7% of the control littermates survived (Figure 2E). The animals were monitored for 2 weeks, and no late deaths were found, indicating that PAVA treatment provides lasting protection. Moreover, PAVA treatment significantly normalized the core body temperature of endotoxemic mice at 12 h and 24 h following LPS challenge (Figure 2F). In contrast, PAVA treatment did not improve the survival rate and core body temperature of *trpv1ko* endotoxemic mice (Figures 2G and 2H). Taken together, these findings indicate that stimulation of TRPV1+ peripheral sensory afferents is an effective method for inhibiting systemic inflammation and protecting the host against lethal inflammation.

### Activation of NTS and C1 neurons in the brainstem via the somatosensory afferents

To determine how PAVA nape s.c. transmit signals to the central nervous system, we used pERK expression to identify activated primary sensory neurons. PAVA nape s.c. significantly increased the number of pERK+ neurons in cervical (C3–C8) DRGs rather than thoracic (T3–T6) DRGs (Figure S3A), indicating that the cervical somatosensory afferents were selectively activated. However, after PAVA administration, the number of pERK+ neurons in NGs was not increased compared to control (Figure S3B). Therefore, neuronal signals activated by PAVA nape s.c. were mainly transmitted to the central nervous system via the cervical peripheral somatosensory afferents.

**Figure 3:**
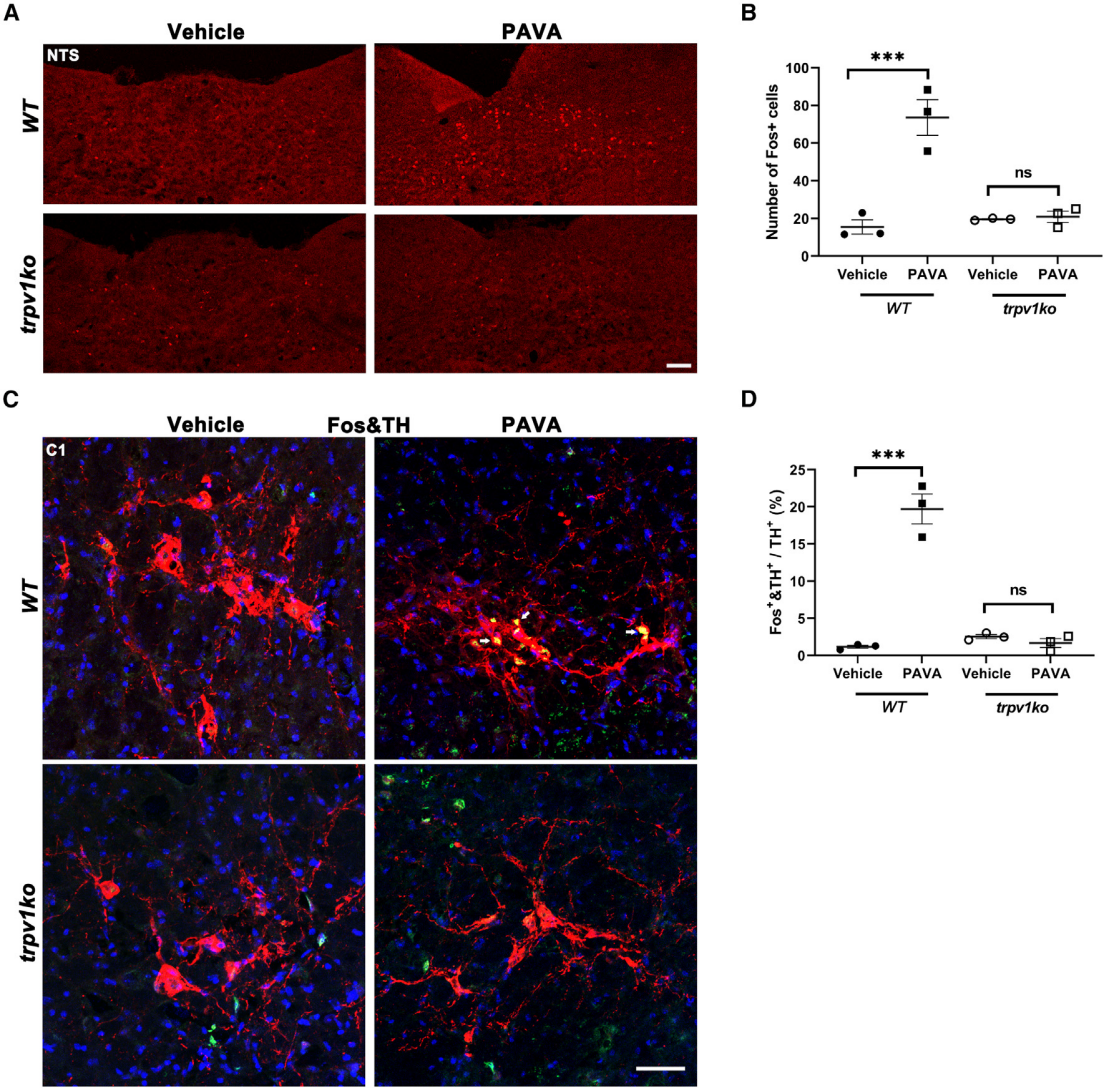
PAVA nape s.c. activated NTS and C1 neurons in the brainstem (A) PAVA nape s.c. induced Fos expression in NTS in wild-type mice but not in *trpv1ko* mice. Scale bar: 100 μm. (B) Quantification of Fos+ neurons in *WT* and *trpv1ko* NTS. (*n* = 3/group). (C) PAVA nape s.c. induced Fos expression (green) in TH+ (red) C1 neurons in wild-type mice but not in *trpv1ko* mice. Arrow, Fos+TH+ C1 neurons. Scale bar: 50 μm. (D) The percentage of Fos expression induced by PAVA treatment in TH + C1 neurons in *WT* and *trpv1ko* mice. (*n* = 3/group). Data are presented as the mean ± SEM, two-way ANOVA with Bonferroni post tests (B and D). ∗∗∗*p* < 0.001; ns, not significant.

To further explore which brainstem nucleus is excited following PAVA treatment, we scanned the expression of Fos, a marker of neuronal activation, in the hindbrain. PAVA nape s.c. strongly induced Fos labeling in NTS compared to control (Figures 3A and 3B). In *trpv1ko* NTS, PAVA injection could not increase Fos+ neurons. NTS receives and integrates both vagal afferents and somatic afferents through spinal ascending pathway, and modulates vagal efferent nerves, and innervates the paraventricular nucleus (PVN) of the hypothalamus, which in turn activates the hypothalamic-pituitary-adrenocortical (HPA) pathway.^22^^,^^23^^,^^24^^,^^25^ NTS plays an important role in controlling peripheral immune responses.^26^ In addition, PAVA administration strongly induced Fos expression in C1 neurons labeled with tyrosine hydroxylase (TH), but this Fos induction was eliminated in *trpv1ko* mice (Figures 3C and 3D). C1 neurons located in the rostral ventrolateral medulla (RVLM) receive visceral and somatic sensory information and serve as a center to control multiple physiological functions. These cells modulate sympathetic efferent pathways.^27^^,^^28^ Furthermore, these neurons also innervate PVN of the hypothalamus to modulate HPA axis. C1 neuron stimulation can activate both the splenic and adrenal sympathetic anti-inflammatory pathways.^11^^,^^29^^,^^30^ Thus, PAVA nape s.c. strongly activated NTS and C1 neurons in the medulla oblongata.

### TRPV1+ peripheral afferent stimulating promoted corticosterone secretion

**Figure 4:**
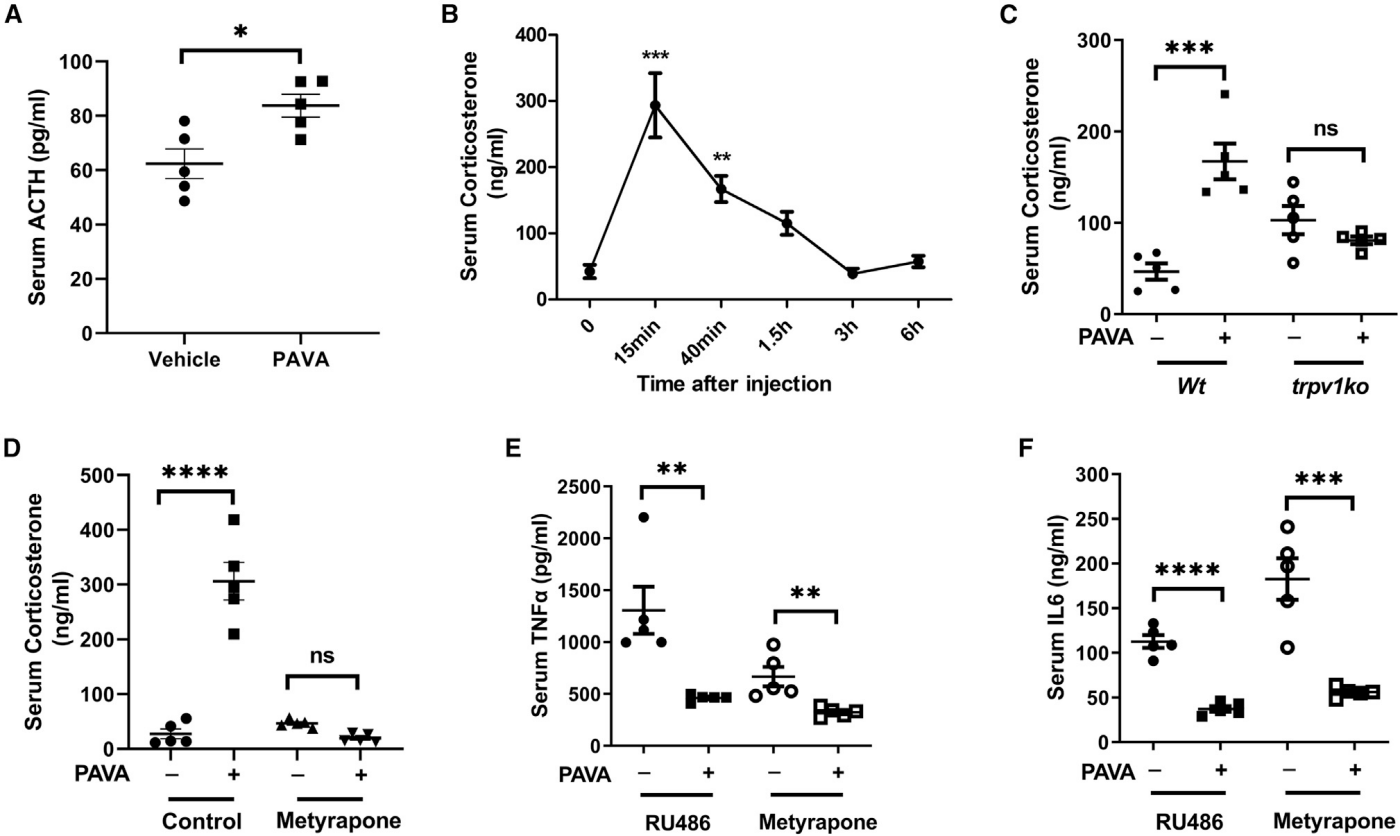
PAVA nape s.c. increased serum level of ACTH and corticosterone (A) PAVA nape s.c. increased serum ACTH level at 5 min after stimulation. (*n* = 5/group). (B) Serum level of corticosterone at different time points following PAVA nape s.c. (*n* = 4–5/time point). (C) PAVA nape s.c. increased serum corticosterone release in wild-type but not *trpv1ko* mice. (*n* = 5/group). (D) Metyrapone pretreatment abolished the PAVA-induced increase of corticosterone. (*n* = 5/group). (E and F) Neither mifepristone (RU486) nor metyrapone pretreatment affected PAVA-evoked anti-inflammatory effects on the expression of TNF-α (E) and IL-6 (F) (*n* = 5/group). Data are presented as the mean ± SEM. Unpaired two-sided Student’s t test (A, E, and F), one-way ANOVA with Bonferroni post tests (B), or two-way ANOVA with Bonferroni post tests (C and D). ∗∗∗∗*p* < 0.0001, ∗∗∗*p* < 0.001, ∗∗*p* < 0.01, ∗*p* < 0.05; ns, not significant.

Activation of hypothalamus promotes the pituitary gland to release adrenocorticotropic hormone (ACTH) into the bloodstream. Circulating ACTH drives the adrenal cortex to secrete glucocorticoids, which have multiple effects on immune cells to inhibit inflammation.^31^ To determine whether PAVA administration activates the HPA axis, we measured the expression of serum ACTH and corticosterone. Serum ACTH level was quickly increased at 5 min following PAVA treatment (Figure 4A). Next, we performed a kinetic analysis of serum corticosterone expression, which indicated that the corticosterone level increased approximately 7-fold at 15 min following PAVA treatment and gradually decreased to baseline within 3 h (Figure 4B). This PAVA-induced increase in serum corticosterone was eliminated in *trpv1ko* mice (Figure 4C). To explore whether the induction of corticosterone modulates the protective effect of PAVA administration, we used metyrapone and mifepristone (RU486) to inhibit the synthesis of corticosterone and block glucocorticoid receptors, respectively.^11^^,^^32^ Metyrapone pretreatment abolished the PAVA-evoked increase of corticosterone (Figure 4D). However, neither treatment affected the anti-inflammatory effect of PAVA treatment on reducing the release of TNF-α and IL-6 (Figures 4E and 4F). The possibility that HPA axis could play a redundant role or affect other immune responses was not ruled out.

### The vagal-adrenal reflex increased circulating catecholamines

**Figure 5:**
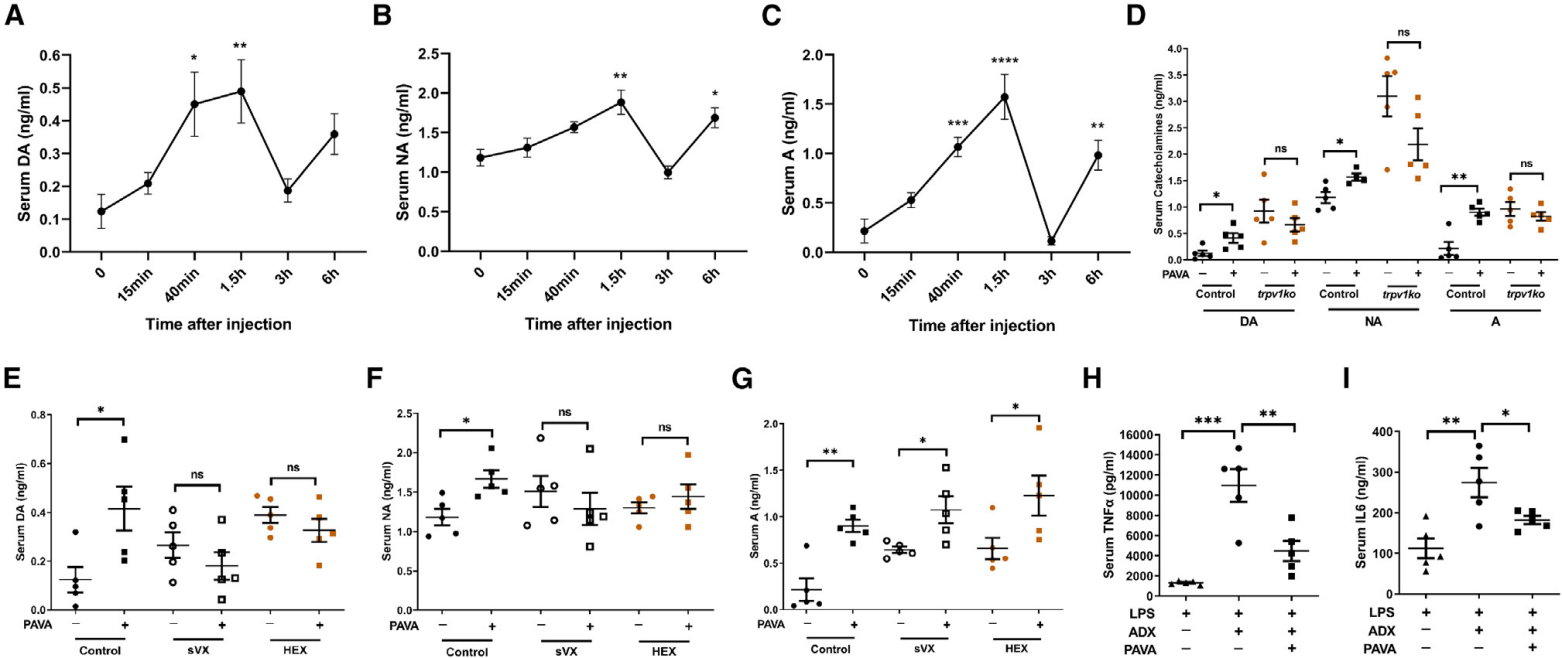
PAVA nape s.c. induced serum catecholamine release (A–C) Serum levels of dopamine (A), noradrenaline (B), and adrenaline (C) at different time points following PAVA nape s.c. (*n* = 4–5/time point). (D) PAVA nape s.c. increased serum catecholamine release in wild-type but not *trpv1ko* mice. (*n* = 4–5/group). (E–G) Both sVX and HEX pretreatment inhibited the release of dopamine (E) and noradrenaline (F) but not adrenaline (G). (*n* = 5/group). (H and I) Adrenalectomy (ADX) did not affect PAVA-evoked anti-inflammatory effects on the expression of TNF-α (H) and IL-6 (I). (*n* = 5/group). Data are presented as the mean ± SEM. One-way ANOVA with Bonferroni post tests (A–C) or unpaired two-sided Student’s t test (D–I). ∗∗∗∗*p* < 0.0001, ∗∗∗*p* < 0.001, ∗∗*p* < 0.01, ∗*p* < 0.05; ns, not significant.

Thermal cutaneous stimulation (>43°C) was reported to promote the secretion of adrenaline and noradrenaline via the autonomic-adrenal axis.^33^ Our kinetic examination of serum catecholamines following PAVA administration revealed increased expression. The levels of all of these molecules, predominantly adrenaline and dopamine, increased significantly at 40 min and 1.5 h after PAVA treatment, decreased to baseline at 3 h, and then increased again by 6 h (Figures 5A–5C). This increase in PAVA-evoked serum catecholamines was eliminated in *trpv1ko* mice (Figure 5D). Serum catecholamines are mainly produced by the chromaffin cells of the adrenal medulla, which are modulated by vagal and sympathetic preganglionic nerves through the release of acetylcholine.^12^^,^^13^^,^^14^^,^^34^ Hexamethonium (HEX) is a nonselective nicotinic cholinergic receptor (nAChR) antagonist. Both subdiaphragmatic vagotomy (sVX) and HEX treatment completely abolished the induction of dopamine and noradrenaline (Figures 5E and 5F), indicating that stimulation of TRPV1+ peripheral sensory afferents can drive the vagal-adrenal reflex to secrete serum catecholamines by signaling through nAChR. However, the induction of adrenaline was not effectively blocked by sVX or HEX treatment (Figure 5G), suggesting that the induction of adrenaline is only partially dependent on the vagal-adrenal reflex. Some reports have indicated that immune cells can also generate and release catecholamines.^35^^,^^36^ Adrenalectomy was performed to abolish the production of circulating catecholamines and corticosterone. Compared with their non-adrenalectomized littermates, the adrenalectomized mice exhibited substantially increased LPS-evoked cytokine production (Figures 5H and 5I), indicating that serum catecholamines and corticosterone are important for the modulation of systemic inflammation. However, adrenalectomy did not affect the anti-inflammatory effects of PAVA treatment (Figures 5H and 5I). Because both circulating catecholamines and autonomic nervous system play crucial roles in modulating multiple physiological functions, we measured vital signs following PAVA treatment. Systolic blood pressure and heart rate were decreased after PAVA nape s.c. injection, suggesting that stimulating TRPV1+ peripheral afferents can affect visceral functions via the somato-autonomic reflex (Figure S4).

### Local catecholamine secretion in the spleen was required for anti-inflammatory effects

**Figure 6:**
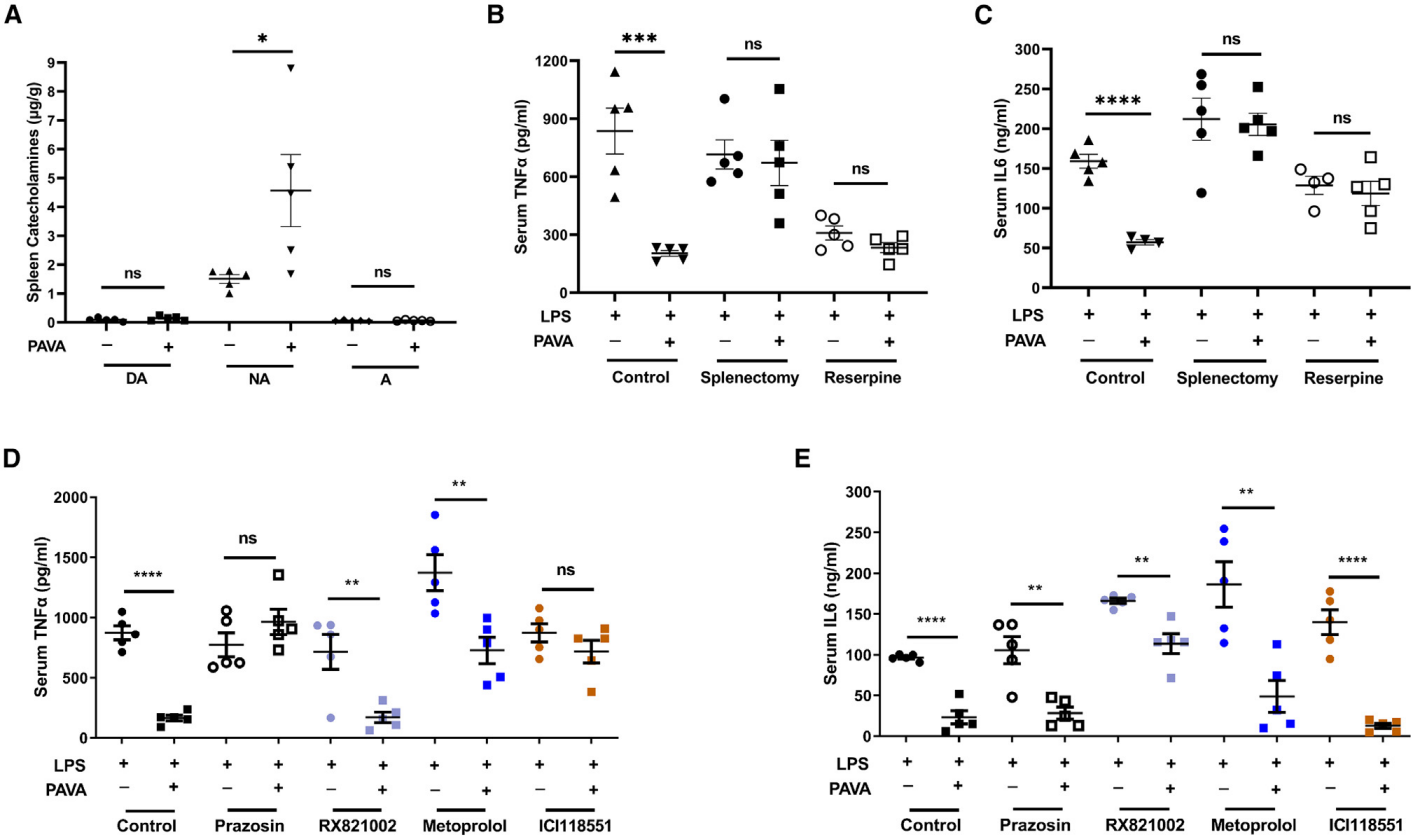
Noradrenaline secretion in the spleen was required for PAVA-induced anti-inflammatory effects (A) PAVA nape s.c. promoted the release of splenic noradrenaline. (*n* = 5/group). (B and C) Serum levels of TNF-α (B) and IL-6 (C) in mice subjected to sham surgery (control), splenectomy or reserpine treatment before PAVA administration and LPS challenge. (*n* = 5/group). (D and E) Noradrenaline regulated serum TNF-α (D) and IL-6 (E) depending on distinct adrenoceptors. (*n* = 5/group). Data are presented as the mean ± SEM. Unpaired two-sided Student’s t test. ∗∗∗∗*p* < 0.0001, ∗∗∗*p* < 0.001, ∗∗*p* < 0.01, ∗*p* < 0.05; ns, not significant.

Both the sympathetic and vagal preganglionic nerves innervate the celiac ganglion (CG) and modulate the postganglionic splenic nerve through the release of acetylcholine.^37^^,^^38^^,^^39^ Activated splenic nerve terminals secrete noradrenaline in the spleen, which in turn inhibits proinflammatory cytokine production.^11^^,^^13^^,^^29^^,^^40^ Our study showed that PAVA administration increased local noradrenaline release in the spleen (Figure 6A). PAVA treatment failed to inhibit the production of TNF-α and IL-6 in splenectomized mice (Figures 6B and 6C), indicating that the spleen plays a critical role in the anti-inflammatory pathway activated by PAVA treatment. Reserpine, a blocker of the vesicular monoamine transporter, was injected i.p. to deplete catecholamine stores in peripheral sympathetic nerve endings.^41^ The anti-inflammatory effect of PAVA treatment was eliminated after the blockade of local noradrenaline release in the spleen (Figures 6B and 6C). To explore which adrenergic receptors may respond to the protective effects of noradrenaline, we tested the effects of the antagonists prazosin, RX821002, metoprolol and ICI118551 to block α1, α2, β1, and β2-adrenoceptors, respectively.^35^ Blockade of the α1- or β2-adrenoceptor abolished the inhibition of TNF-α production but did not affect the release of IL-6 (Figures 6D and 6E). We speculated that distinct adrenergic receptors regulate the production of some cytokines in a redundant manner, in which the anti-inflammatory effects are not abrogated by blocking one of them. Thus, noradrenaline released from splenic nerve terminals mediated the anti-inflammatory effects of PAVA treatment.

### TRPV1+ peripheral nerve stimulating mediated gene expression in the spleen

**Figure 7:**
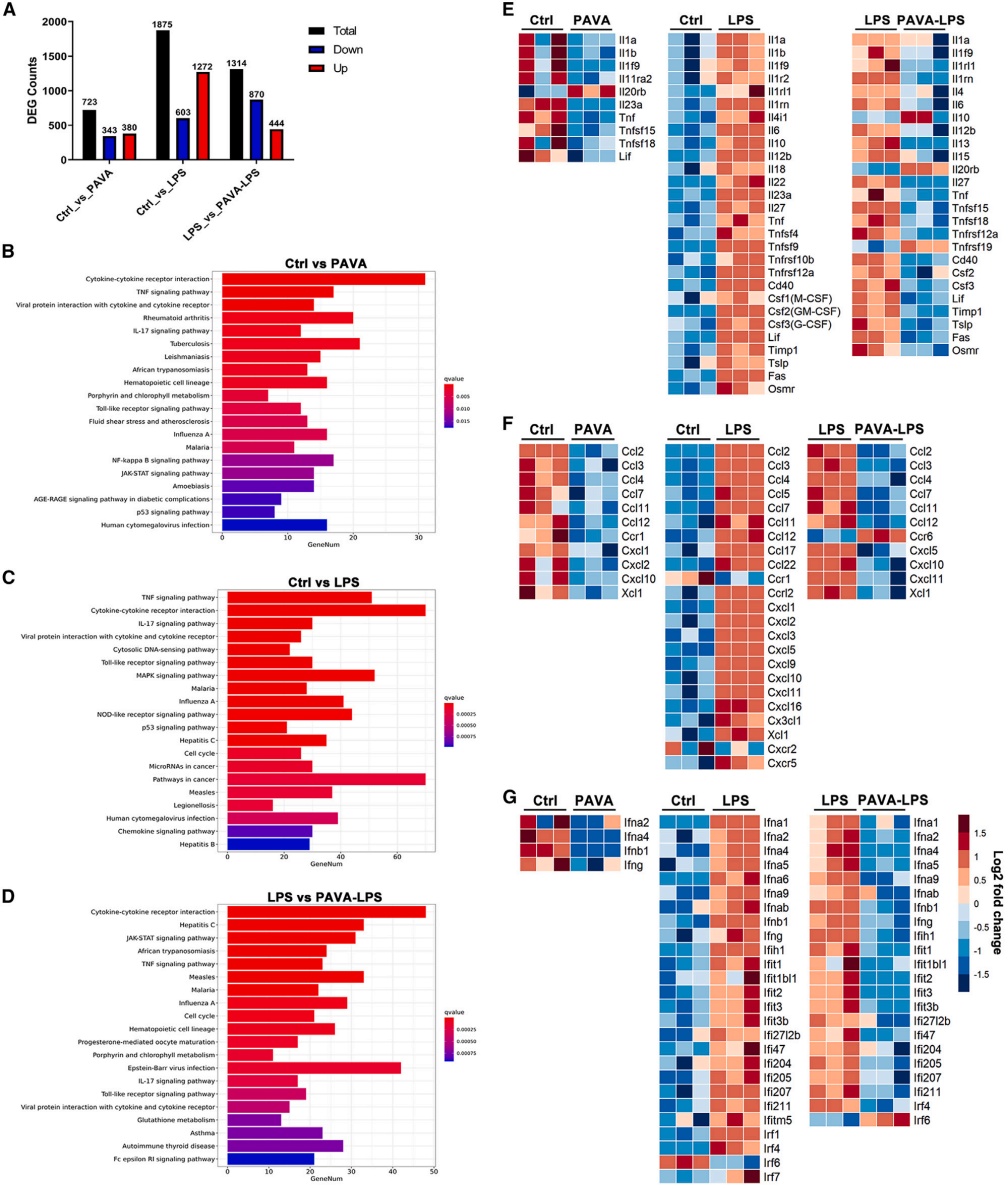
RNA-seq analysis revealed that PAVA treatment induced significant alterations in gene expression in the spleen (A) The number of DEGs induced by distinct treatments. (B–D) KEGG analysis showing enriched pathways for comparing control group to PAVA only group (B), comparing control group to LPS only group (C), and comparing LPS only group to PAVA-LPS group (D). (E–G) Heatmap showing the RNA levels of splenic cytokines (E), chemokines (F), and interferon-related genes (G) at 1 h following indicated treatments. The scale in the heatmaps shows FPKM values transformed to log_2_(FPKM) values for color scaling.

RNA-seq analysis was performed to characterize the transcriptional changes in the spleen triggered by distinct treatments, including saline control, PAVA alone, LPS alone or PAVA-LPS treatment (Figure 7A). Functional pathway analysis revealed that differentially expressed genes (DEGs) were enriched in cytokine-cytokine receptor interaction, TNF, IL-17, and Toll-like receptor signaling pathways in each of the comparisons (Figures 7B–7D). PAVA treatment restrained the expression of some proinflammatory genes, such as *map2k3* and *pik3r3*, which play key roles in multiple signaling pathways to promote cytokine production (Table S1). Consistent with our Luminex analysis of serum protein levels, the mRNA levels of a large group of proinflammatory cytokines were substantially increased in the spleen following LPS challenge, while PAVA pretreatment inhibited most of these cytokines and increased anti-inflammatory IL-10 level (Figures 7E–7G). In addition, RNA-seq data showed that treatment with PAVA alone significantly altered gene expression in spleen. Compared to that in the control littermates, the downregulation of proinflammatory cytokines in the PAVA-treated mice was most notable (Figures 7E–7G). PAVA treatment not only induced anti-inflammatory effects in inflamed states but also inhibited the immune response under normal physiological conditions.

### Autonomic-immune reflex was activated to suppress inflammation

**Figure 8:**
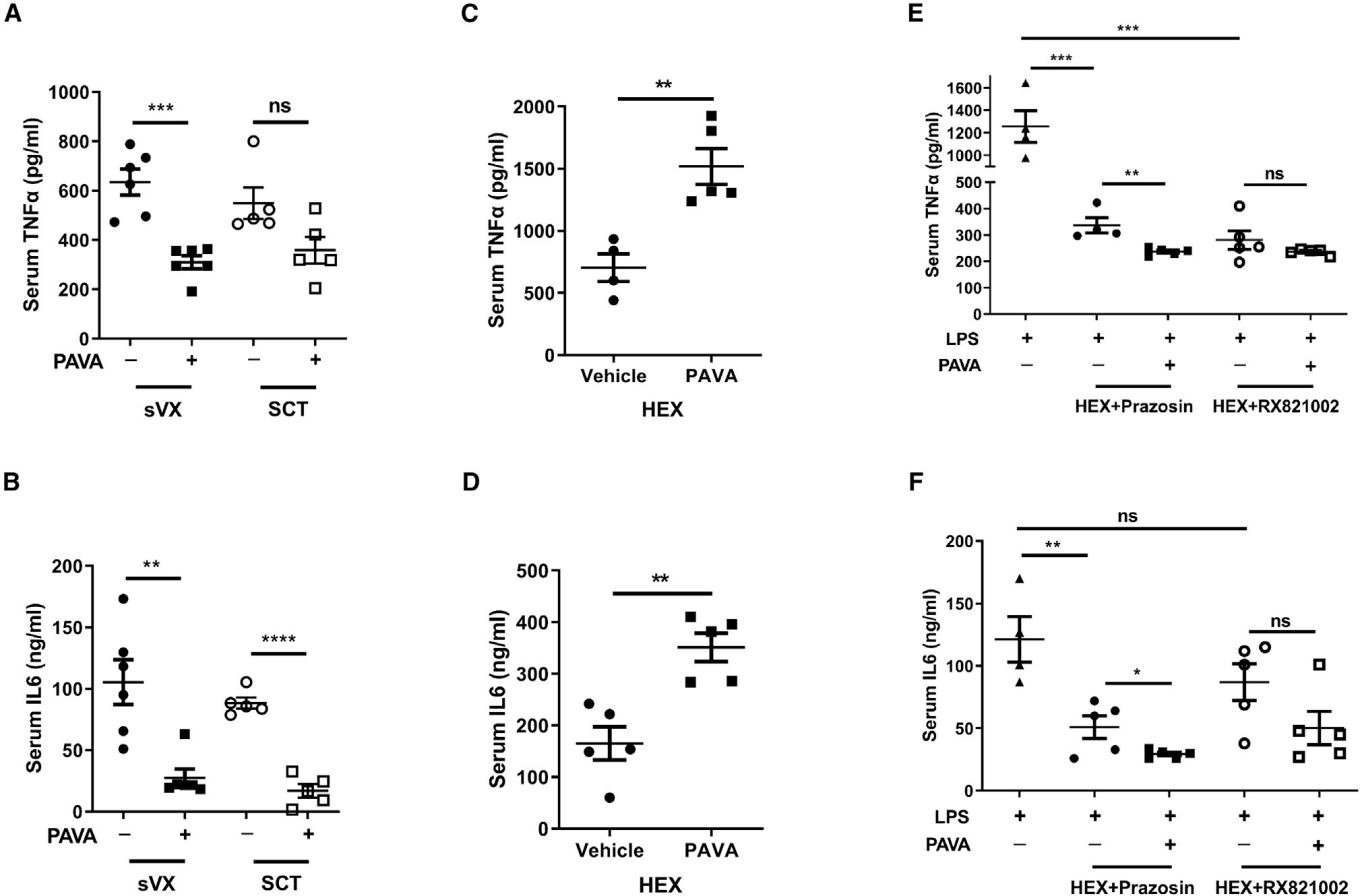
Stimulation of TRPV1+ peripheral afferents activated the autonomic-splenic axis to suppress inflammatory cytokine production (A and B) Serum TNF-α (A) and IL-6 (B) in mice treated with sVX or T2 SCT before PAVA administration and LPS challenge. (*n* = 5–6/group). (C and D) Serum TNF-α (C) and IL-6 (D) were increased with HEX pretreatment before PAVA administration and LPS challenge. (*n* = 4–5/group). (E and F) Serum TNF-α (E) and IL-6 (F) in the mice treated with HEX+prazosin or HEX+RX821002 before PAVA administration and LPS challenge. (*n* = 4–5/group). Data are presented as the mean ± SEM. Unpaired two-sided Student’s t test. ∗∗∗∗*p* < 0.0001, ∗∗∗*p* < 0.001, ∗∗*p* < 0.01, ∗*p* < 0.05; ns, not significant.

To identify which division of the autonomic-immune axis mediates the anti-inflammatory effect of PAVA treatment, we performed sVX to block both the vagal-adrenal and vagal-CG-splenic axes. However, sVX did not affect the anti-inflammatory effects of PAVA treatment (Figures 8A and 8B). Previous studies demonstrated that α7 nicotinic acetylcholine receptor (α7nAChR) was present in the murine CG and spleen, and played a crucial role in the cholinergic anti-inflammatory pathway induced by vagus nerve stimulation.^42^^,^^43^^,^^44^ We next investigated the effect of methyllycaconitine citrate (MLA), a selective α7nAChR antagonist.^45^ Although MLA pretreatment suppressed LPS-induced TNF-α production, it did not abolish the inhibition of IL-6 release (Figure S5). Blocking α7nAChR is not enough to eliminate the anti-inflammatory effects of PAVA treatment. Spinal cord transection (SCT) at thoracic level 2 (T2) abrogates the sympathetic-splenic reflex.^46^ T2 SCT eliminated the inhibition of TNF-α but did not affect IL-6 expression (Figures 8A and 8B), suggesting that the anti-inflammatory effects induced by PAVA treatment are partially dependent on the sympathetic efferent pathway originating from the supraspinal (medullary) reflex. HEX, a ganglionic blocker that inhibits nicotinic cholinergic ganglionic neurotransmission, inhibits both sympathetic and vagal preganglionic nerves to activate the splenic sympathetic nerve.^47^ In addition, this treatment suppressed the release of serum dopamine and noradrenaline induced by PAVA administration (Figures 5E and 5F). Surprisingly, following HEX treatment, PAVA administration substantially increased the LPS-evoked release of TNF-α and IL-6 (Figures 8C and 8D), suggesting that TRPV1+ peripheral afferent stimulation activates both the sympathetic and vagal efferent pathways to suppress inflammation. HEX pretreatment switched the effect of PAVA treatment from an anti-inflammatory effect to a proinflammatory effect. It suggested that stimulating TRPV1+ peripheral nerves at the nape induced a composite immune response that causes a strong autonomically mediated anti-inflammatory effect and a weaker proinflammatory action masked by the anti-inflammatory functions. Previous studies have indicated that serum adrenaline promotes the inflammatory cascade by activating α-adrenoceptors.^35^^,^^36^ Our studies showed that HEX treatment did not block the increase in serum adrenaline induced by PAVA administration (Figure 5G). After the mice were treated with HEX in combination with prazosin or RX821002, the LPS-evoked production of TNF-α and IL-6 decreased compared to that observed in the mice treated with LPS only, HEX-LPS, prazosin-LPS and RX821002-LPS. In addition, PAVA treatment regained the ability to suppress the release of TNF-α and IL-6 in the HEX-prazosin pretreated mice (Figures 8E and 8F), suggesting that PAVA-induced secretion of serum adrenaline promotes inflammatory cytokine production by activating α-adrenoceptors. In contrast to the results in the HEX-treated mice, PAVA administration did not increase cytokine expression in the reserpine-treated or splenectomized mice (Figures 6A and 6B), suggesting that serum dopamine and noradrenaline induced via the vagal-adrenal axis are also involved in inhibiting inflammation. Taken together, these findings indicate that stimulating TRPV1+ peripheral afferents activates both sympathetic-immune and parasympathetic-immune reflexes to synergistically suppress systemic inflammation.

## Discussion

Cumulative evidence supports the idea that the somatosensory nervous system not only identifies and transmits sensory information from the skin and internal tissues but also regulates peripheral immune responses.^48^^,^^49^ The anti-inflammatory effects of peripheral somatosensory afferent stimulation are dependent on specific stimulation sites. Electric stimulation at the hindlimb ST36 acupoint activates dopamine-induced anti-inflammatory effects through vagal-adrenal axis.^12^^,^^14^ Electroacupuncture at the abdomen ST25 acupoint elicits splenic nerve endings to release noradrenaline via spinal-splenic sympathetic reflex, which in turn suppress inflammation.^13^ Among our treatments, stimulating TRPV1+ peripheral nerves at the nape was the most effective method for suppressing cytokine production. It activated the HPA axis to rapidly secrete corticosterone, drove the vagal-adrenal axis to generate serum catecholamines, and provoked autonomic-splenic axis to suppress cytokine production. Through the somato-autonomic reflex, both the sympathetic and parasympathetic efferent pathways were activated to synergistically induce anti-inflammatory effects. In addition, TRPV1+ peripheral nerve stimulation at the nape activated NTS and C1 neurons in the brainstem. T2 SCT partially eliminated the anti-inflammatory effects. Taken together, these findings indicate that stimulating TRPV1+ peripheral nerves at the nape suppress systemic inflammation via the supraspinal (medullary) reflex in which the brain receives sensory inputs and drives sympathetic and parasympathetic efferent pathways to modulate physiological functions.

Spleen-innervating primary sensory neurons are TRPV1+ nociceptors predominantly located in left T8–T13 DRGs ipsilateral to the spleen.^50^ In spinal cord, spleen-innervating neurons are consistent with the location of the left sympathetic preganglionic neurons at T4–T9 spinal levels.^46^ Somatosensory information from the trunk and limbs is transmitted to the same or a nearby segment spinal cord where the sympathetic preganglionic nerves are elicited to modulate visceral functions. This somato-spinal sympathetic reflex has a strong segmental organization.^13^^,^^51^ Dietary capsaicin can activate TRPV1+ nociceptors in left T8–T13 DRGs to enhance the splenic germinal center response and humoral immunity.^50^ In the present study, it seems possible that stimulating TRPV1+ somatic afferents at the abdomen or the back excite TRPV1+ nociceptors in T8–T13 DRGs, which in turn activate a segmental spinal sympathetic reflex to suppress cytokine production in the spleen. Future studies will explore which anti-inflammatory circuits are activated by stimulating TRPV1+ somatic afferents at the abdomen and the back.

A number studies demonstrated that electric stimulation of vagal nerves and the dorsal motor nuclei of the vagus can attenuate inflammation, indicating that vagus afferent and efferent nerves are critical for modulating innate immunity.^10^^,^^11^^,^^52^ Previous studies and our study showed that stimulating TRPV1+ visceral sensory afferents (PAVA i.p.) could inhibit TNF-α production (Figure 1B), while ablation of TRPV1+ vagal sensory neurons in NG promoted inflammation during bacterial infection, suggesting that TRPV1+ visceral somatosensory afferents and vagal sensory afferents play crucial roles in controlling immune response.^53^^,^^54^^,^^55^^,^^56^^,^^57^ Recent study demonstrated that activation of TRPA1+ or CALCA+ vagal sensory neurons could elicit NTS to induce anti-inflammatory effects.^26^ Both TRPA1 and CALCA highly overlap with TRPV1 expression in NG.^9^ We propose that TRPV1+ vagal afferents can identify increased inflammatory signals via distinct inflammatory mediator receptors expressed on nerves and subsequently activate the brain-visceral interactions to protect organs from excessive inflammation. This vagal-autonomic reflex, including vagal-sympathetic and vagal-vagal reflexes, is critical for monitoring organismal inflammatory state and rebalancing internal immune response. Accordingly, we anticipate that disruption of this reflex will lead to out-of-control of immune response and induce a wide range of inflammatory and autoimmune diseases. Therefore, both somato-autonomic and vagal-autonomic reflexes are crucial for maintaining immune homeostasis and modulating immune responses.

A comprehensive adaptable response of the host to excessive external stimuli via the neural-visceral reflex is crucial for stress resistance and survival. Here, we found that stimulation of TRPV1+ peripheral somatosensory afferents modulated immune responses and visceral functions, such as the secretion of serum corticosterone and catecholamines, blood pressure, and heart rate. Excessive external stimuli may affect internal homeostasis via somato-autonomic and somato-hormonal reflexes and neuroimmune communication. It is noteworthy that injury and pathogen infection in peripheral tissues excite nociceptors, pruriceptors, or thermoreceptors, which may transiently or continuously affect immunity and internal organ function. Our study revealed a specific neural circuit that stimulating TRPV1+ somatosensory afferents at the nape could concurrently drive both sympathetic and parasympathetic efferent pathways to synergistically induce anti-inflammatory effects. Stimulation intensity, somatosensory neuron specificity and body region specificity are all crucial for stimulating somatosensory fibers to modulate immune responses. Otherwise, treatment may cause complex or unwanted adverse reactions, such as stimulation of MRGPRD+ afferents, or may be insufficient to achieve the desired effects. Due to this endogenous mechanism, stimulation of TRPV1+ peripheral sensory afferents in specific body areas provides an effective strategy for preventing and treating inflammatory diseases.

### Limitations of the study

Previous studies reported that male and female mice show differently sensitivity to LPS.^13^^,^^52^ Therefore, this study focused on male mice. Female mice will be studied in future investigations to develop appropriate therapeutic approach for different gender. PAVA nape s.c. decreased systolic blood pressure and heart rate, which would be undesirable side effects in future clinical application. Although it seems possible that stimulating TRPV1+ peripheral afferents can affect visceral functions via the somato-autonomic reflex, this requires further investigation. Future studies are needed to explore whether appropriate stimulation intensity and specific stimulation position can treat systemic inflammation without affecting the blood pressure and heart rate. In addition, future studies are also needed to evaluate the therapeutic potential for TRPV1+ peripheral nerve activation in hypertensive patients.

## Resource availability

### Lead contact

Further information and requests for resources and reagents should be directed to the lead contact, Zijing Liu (liuzijing@hotmail.com).

### Materials availability

This study did not generate new unique reagents.

### Data and code availability

• RNA-seq data have been deposited at GEO and are publicly available as of the date of publication. Accession number is listed in the key resources table.
• This paper does not report original code.
• Any additional information required to reanalyze the data reported in this paper is available from the lead contact upon request.

## Acknowledgments

We thank Qiang Sun and Nannan Du from Beijing institute of biotechnology for technical assistance in measuring blood pressure and heart rate of the mice. Graphical Abstract is created with BioGDP.com.^58^ This study is supported by the 10.13039/501100001809National Natural Science Foundation of China (31371102).

## Author contributions

D.S., Z.C., and F.M. conducted the experiments; Z.L. and Y.H. created the figures; C.C. analyzed the data and supervised the study; Z.L. designed and supervised the entire study, analyzed the data, and wrote the manuscript.

## Declaration of interests

C.C. and Z.L. have a patent application related to this work.

## STAR★Methods

### Key resources table

**Table:** 

REAGENT or RESOURCE	SOURCE	IDENTIFIER
**Antibodies**		

Rabbit anti-pERK	Cell Signaling Technology	Cat#4370S
Rabbit anti-c-Fos	Santa Cruz	Cat#SC-52
Rabbit anti-TH	Millipore	Cat#AB152
Alexa Fluor 488-conjugated goat anti-rabbit antibody	Invitrogen	Cat#A11008
Alexa Fluor 568-conjugated goat anti-rabbit antibody	Invitrogen	Cat#A11011

**Chemicals, peptides, and recombinant proteins**		

Lipopolysaccharide	Sigma	Cat# L2630
β-alanine	Sigma	Cat# A9920
Hexamethonium bromide	Sigma	Cat# H0879
ICI118551	Sigma	Cat# I127
Reserpine	Sigma	Cat# 83580
RX821002	Sigma	Cat# R9525
Dexamethasone	MedChemExpress	Cat# HY-14648
Metoprolol	MedChemExpress	Cat# HY-17503A
Metyrapone	MedChemExpress	Cat# HY-B1232
Methyllycaconitine citrate	MedChemExpress	Cat# HY-N2332A
Mifepristone	MedChemExpress	Cat# HY-13683
Prazosin	MedChemExpress	Cat# HY-B0193A
Resiniferatoxin	MedChemExpress	Cat# HY-N2333
Nonivamide	Apexbio	Cat# A3278
Capsaicin cream	Changchun Puhua	Cat# H20030031
Trpv1 probe	ACD	Cat# 313331

**Critical commercial assays**		

TNFα (mouse) ELISA kit	Multisciences	Cat# EK282/4-96
IL-1β (mouse) ELISA kit	R&D Systems	Cat# MLB00C
IL-6 (mouse) ELISA kit	R&D Systems	Cat# M6000B
Parameter Corticosterone Assay kit	R&D Systems	Cat# KGE009
Mouse/Rat ACTH ELISA kit	Abcam	Cat# ab263880
Bio-Plex Pro Mouse Chemokine Panel 31-Plex	Bio-Rad	Cat# 12009159
3-CAT ELISA kit	Labor Diagnostika Nord	Cat# BA E-5600R
TSA plus Fluorescein	AKOYA	Cat# NEL741001KT
TSA plus Cy3	AKOYA	Cat# NEL744001KT
RNAscope Multiplex Fluorescent Reagent Kit	ACD	Cat# 323100
RNA Protein Co Detection Assays	ACD	Cat# 323180

**Deposited data**		

RNAseq data of the spleen	This paper	GEO: GSE264378

**Experimental models: Organisms/strains**		

Mouse: C57BL/6J	Beijing Vital River Laboratory Animal Technology	219
Mouse: Trpv1^-/-^	Cyagen Biosciences	S-KO-03959

**Software and algorithms**		

GraphPad Prism	GraphPad Software	Version 8.0.2

**Other**		

Zeiss LSM 800 confocal microscope	Zeiss	N/A
Blood pressure system	Softron Biotechnology	BP-2010A

### Experimental model and study participant details

#### Animals

C57BL/6J male mice were purchased from Beijing Vital River Laboratory Animal Technology Co. *Trpv1-/-* mice (S-KO-03959) were obtained from Cyagen Biosciences. Mice were maintained on a 12h light/dark cycle in a standard barrier environment with free access to food and water. Age-matched 6-11 week old male mice were used for experiments. All animal experiments were approved by the Institutional Animal Care and Use Committee of Beijing Institute of Biotechnology (IACUC-DWZX-2023-050).

### Method details

#### Drugs treatment

Lipopolysaccharide (LPS) (L2630), β-alanine (A9920), hexamethonium bromide (HEX) (H0879), ICI118551 (I127), reserpine (83580) and RX821002 (R9525) were purchased from Sigma-Aldrich. Dexamethasone (HY-14648), metoprolol (HY-17503A), metyrapone (HY-B1232), methyllycaconitine citrate (MLA) (HY-N2332A), mifepristone (HY-13683), prazosin (HY-B0193A) and resiniferatoxin (RTX) (HY-N2333) were purchased from MedChemExpress.

Nonivamide or Pelargonic acid vanillylamide (PAVA, A3278, Apexbio) was dissolved in 7% Tween-80 diluted in sterile saline. PAVA (4 mg/kg) was administered intraperitoneally (i.p.) 30 min before LPS injection. PAVA (4 mg/kg) was administered subcutaneously (s.c.) into the nape 30 min before LPS injection. PAVA (4 mg/kg) was administered s.c. into the abdomen 30 min before LPS injection. PAVA (4 mg/kg) was administered s.c. into the back close to the tail 30 min before LPS injection. PAVA (0.25 mg/kg) was administered intravenously (i.v.) into the tail veins 30 min before LPS injection. Capsaicin cream (0.25 mg/g, H20030031) was purchased from Changchun Puhua Co. PAVA dissolved in Tween-80 was mixed with capsaicin cream (0.25 mg/g) to produce CAP cream (PAVA 0.75 mg/g + capsaicin 0.25 mg/g). CAP cream was applied topically to the shaved nape 30 min before endotoxemia challenge. β-alanine (5 mg/kg or 25 mg/kg) was injected s.c. into the nape 30 min before endotoxemia challenge. Dexamethasone (5 mg/kg) were injected i.p. 30 min before LPS challenge. Prazosin (2 mg/kg), RX821002 (10 mg/kg), metoprolol (15 mg/kg), ICI118551 (8 mg/kg), HEX (10 mg/kg), mifepristone (30 mg/kg), and MLA (6 mg/kg) were injected i.p. 60 min before LPS challenge.^11^^,^^29^^,^^35^^,^^45^ Metyrapone (150 mg/kg) was administered s.c. into the abdomen 60 min before LPS injection.^32^ Reserpine (10 mg/kg) was administered i.p. 24 h before LPS injection.^12^^,^^41^

#### Endotoxemia model

LPS (8 mg/kg) was injected i.p. into male mice. The sera and spleens of 6- to 8-week-old male mice were collected at the indicated time points. For survival experiments, 9- to 11-week-old male mice were used. If 20% of the body weight was lost, the mice were defined as dead and were euthanized. Weight, core body temperature and clinical symptom score were assessed at the indicated time points. The severity of piloerection, conjunctivitis, diarrhea and lethargy was scored on a three-point scale by two independent researchers.^13^

#### Chemical ablation of TRPV1+ neurons

RTX was dissolved in 2% DMSO with 0.15% Tween 80 in PBS. 4-week-old C57BL/6J male mice were injected s.c. in the flank on consecutive days with three increasing doses of RTX (30, 70, and 100 μg/kg). Control mice were treated with vehicle alone. Mice were rested for 4 weeks before experiments.^54^

#### Thermal treatment

Before the experiment, the nape of each mouse was shaved under anesthesia. The mice were laced on a rodent operating table. An ∼50°C hand warmer (Feixiang) or ∼0°C wet ice was attached to the shaved nape for 15 min. A hand warmer at ∼20°C was attached to the shaved nape for 15 min as the sham control. LPS challenge was performed 15 min after thermal treatment.

#### Surgeries

Mice were handled on a heating rodent operating table to maintain body temperature. All mice were anesthetized with inhaled isoflurane (2%-5%) during the surgical procedures.

#### Adrenalectomy

Anesthetized mice were subjected to dorsal incision to expose the adrenal glands. Both adrenal glands were removed, and the incision was closed using sutures. Adrenalectomized mice were given drinking water supplemented with 0.9% NaCl.^11^

#### Splenectomy

Anesthetized mice were subjected to an abdominal incision to expose the stomach. The stomach was gently retracted to the side to expose the spleen. The spleen was removed, and the incision was closed using sutures.^12^^,^^13^

#### Subdiaphragmatic vagotomy (sVX)

Anesthetized mice were subjected to an abdominal midline incision to expose the stomach. The stomach was gently pulled down to expose the esophagus. The anterior and posterior trunks of the vagal nerves were identified on both sides of the esophagus between the diaphragm and gastric cardia. Then, both vagal branches were transected. The incision was closed using sutures.^12^^,^^13^

#### Spinal cord transaction (SCT)

Anesthetized mice were subjected to laminectomy to expose the dorsal spinal cord. Complete spinal transection was performed with iridectomy scissors at vertebral level T2, and the wound was closed using sutures. After surgery, the mice were injected s.c. with 1 ml of sterile saline. Manual bladder expression was performed twice daily.^46^

#### Blood pressure and heart rate measurement

The blood pressure and heart rate were measured via the noninvasive tail-cuff method (BP-2010A blood pressure system, Softron Biotechnology, Beijing, China). The measurements were performed before and after PAVA treatment. Blood pressure and heart rate values were derived from an average of multiple measurements per animal.

#### Hotplate test

All mice were sequentially placed on a 52°C hot plate, and the latency of hindpaw flicking/licking/jumping was recorded. The cutoff time was set to 45 s to avoid tissue injury.^8^

#### Cytokine analyses

Blood samples were collected at the indicated time points and allowed to clot for 2 h at room temperature before centrifugation for 20 min at 2000 g. Serum was collected and stored at -80°C before use. IL-1β (R&D Systems MLB00C), IL-6 (R&D Systems M6000B) and TNFα (Multisciences EK282/4-96) enzyme-linked immunosorbent assay (ELISA) kits were used according to the manufacturers’ protocols. Luminex assays were performed on a Luminex 200 with a Bio-Plex Pro Mouse Chemokine Panel 31-Plex (12009159, Bio-Rad) following the manufacturer’s instructions.

#### ACTH, catecholamine and corticosterone analysis

PAVA (4 mg/kg) was administered s.c. into the nape, then blood samples were collected at distinct time points. 0.9% Saline or metyrapone (150 mg/kg) was administered s.c. into the abdomen 30 min before PAVA injection, then blood samples were collected at 15 min after PAVA injection. Blood samples were collected between 1 and 5 PM and allowed to clot for 2 h at room temperature before centrifugation for 20 min at 2000 g. Serum was collected and stored at -80°C before use.

For measuring splenic catecholamine concentration, spleens were collected in solution containing EDTA (1mM) and sodium metabisulfite (4mM) at 15min after PAVA treatment. Samples were homogenized and supernatants were collected by centrifugation at 10000 rpm for 10 min. Supernatants were stored at -80°C before use.

Serum ACTH level was measured with the Mouse/Rat ACTH ELISA kit (Abcam ab263880). Catecholamines were measured by a 3-CAT ELISA kit (LDN BA E-5600R). The serum corticosterone concentration was measured with a Parameter Corticosterone Assay kit (R&D Systems KGE009).

#### Immunofluorescence staining

For analysis of pERK induction in NG and DRG, 4 mg/kg PAVA was administered s.c. into the nape. Fifteen minutes later, the mice were euthanized. For Fos analysis in the brainstem, 4 mg/kg PAVA was administered s.c. into the nape. Two hours later, the mice were euthanized. The mice were perfused with 4% paraformaldehyde (PFA) in 1x PBS. The DRGs and NGs were dissected and postfixed in 4% PFA for 2 h at 4°C. The brains were dissected and postfixed in 4% PFA overnight at 4°C. Then, these tissues were dehydrated in 20% sucrose overnight at 4°C and embedded in OCT. The sections were cut at thickness of 12 μm (for DRGs and NGs) and 20 μm (for brains).^8^ The serial coronal sections of the brainstem (bregma -7.10 to -7.90 mm) were chosen for Fos analysis in NTS. For immunofluorescence staining, the sections were blocked with 0.3% Triton X-100 plus 5% goat serum in PBS for 1 h at room temperature and then incubated with diluted primary antibodies overnight at 4°C. After washes with 1x PBS, the sections were incubated with diluted secondary antibodies for 1.5 h at room temperature. Images were acquired with a Zeiss confocal microscope (Zeiss LSM 800). The following antibodies were used: rabbit anti-pERK (1:500, 4370S, Cell Signaling Technology), rabbit anti-c-Fos (1:200, SC-52, Santa Cruz), rabbit anti-TH (1:1000, AB152, Millipore), Alexa Fluor 488-conjugated goat anti-rabbit antibody (1:1000, A11008, Invitrogen), Alexa Fluor 568-conjugated goat anti-rabbit antibody (1:1000, A11011, Invitrogen), TSA plus Fluorescein (1:1000, NEL741001KT, AKOYA), and TSA plus Cy3 (1:1000, NEL744001KT, AKOYA). RNAscope combined with immunofluorescence staining was performed with an RNAscope multiplex fluorescent detection reagent kit (323100, ACD) and an RNA-Protein codetection kit (323180, ACD) following the manufacturer’s protocol. A Trpv1 (313331, ACD) probe was used. Four to eight sections were analyzed for each sample, with 3 adult mice per group. Representative data from experiments that were replicated at least three times with similar results are shown.

#### RNA sequencing (RNA-seq)

For the LPS and PAVA-LPS groups, sterile saline or 4 mg/kg PAVA was administered s.c. into the nape 30 min before LPS injection. Spleens were dissected 1 h after LPS challenge. For the control and PAVA groups, sterile saline or 4 mg/kg PAVA was administered s.c. into the nape, and the spleens were dissected 1 h later. Total RNA was extracted with TRIzol reagent (Life Technologies). The RNA concentration and purity were measured with NanoDrop 2000. A total of 1 μg of RNA per sample was used for RNA sample preparation. Sequencing libraries were generated using the Hieff NGS Ultima Dual-mode mRNA Library Prep Kit for Illumina (Yeasen Biotechnology) following the manufacturer’s instructions, and index codes were added to attribute sequences to each sample. The libraries were sequenced on an Illumina NovaSeq platform to generate 150 bp paired-end reads. Clean data were obtained by removing reads containing adapters, reads containing ploy-N sequences and low-quality reads from the raw data. Clean reads were aligned to the mouse genome (Mus_musculus GRCm38) with HISAT2 tools. Gene expression levels were quantified as fragments per kilobase of transcript per million fragments mapped. Genes with an adjusted P- value < 0.01 and a fold change ≥ 2 according to DESeq2 were considered differentially expressed. We thank Biomarker Technologies Co., Ltd. (Beijing, China) for assisting in sequencing and bioinformatics analysis.

### Quantification and statistical analysis

The data are presented as the mean ± SEM. Biological replicates and individual experiments or ‘n’ represent separate individual mice. Statistical analyses were performed using GraphPad Prism 8 software. Unpaired two-sided Student’s *t*-test was used to compare the mean values between two groups. One-way ANOVA or two-way ANOVA followed by a Bonferroni post hoc correction was used to compare multiple groups. Survival rate analyses were performed using the Mantel-Cox log-rank test. For survival studies and core body temperature measurements, experiments were performed at least three times, and data from individual mice were pooled from all experiments. *p* < 0.05 was considered to indicate statistical significance. ∗∗∗∗*p* < 0.0001, ∗∗∗*p* < 0.001, ∗∗*p* < 0.01, ∗*p* < 0.05; ns, not significant. The statistical details of experiments can be found in the figure legends.

## Supplemental information

Document S1. Figures S1–S5

Table S1. LPS vs. PAVA-LPS KEGG pathway enrichment analysis, related to Figure 7 Output of KEGG enrichment analysis on DEGs.
