# Supplementary material for: Stimulation of TRPV1+ peripheral somatosensory nerves suppress inflammation via the somato-autonomic reflex
Source: iScience. 2025 Jan 17;28(2):111831. doi: 10.1016/j.isci.2025.111831 (PMC11834129; doi:10.1016/j.isci.2025.111831)
Supplement: Document S1. Figures S1–S5 [file mmc1.pdf]

iScience, Volume *28*

Supplemental information

Stimulation of TRPV1+ peripheral somatosensory nerves suppress inflammation via the somato-autonomic reflex

Dengcen Song, Zheng Cao, Yong Hu, Fengyu Mao, Cheng Cao, and Zijing Liu

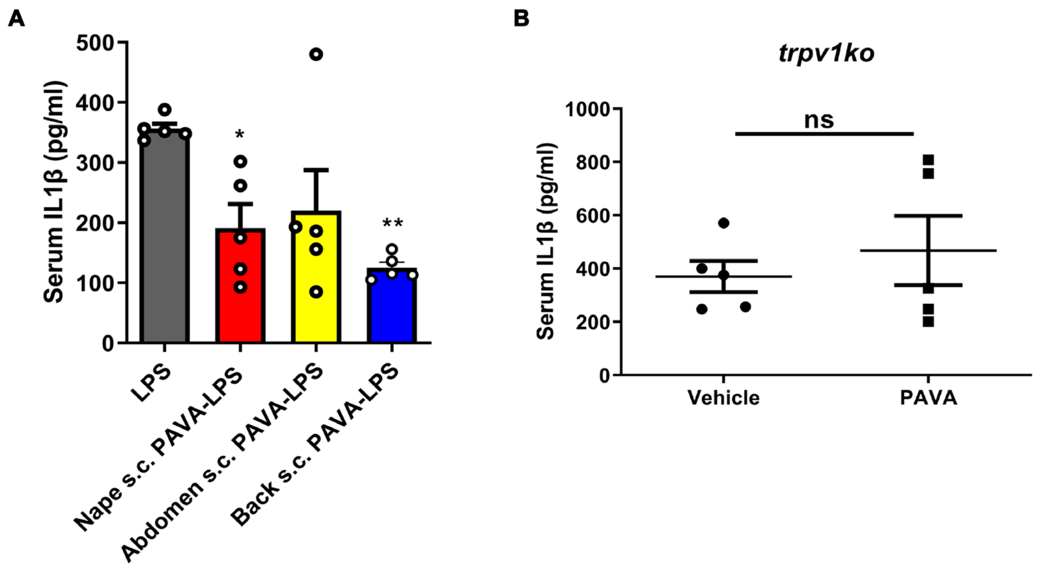

Figure S1. Stimulation of TRPV1+ peripheral sensory afferents suppressed IL1β production, related to Figure 1 and Figure 2.

(A) Serum level of IL1β following distinct PAVA treatments at 6 h after LPS challenge. (n = 5/group) One-way ANOVA with Bonferroni post tests.

(B) PAVA nape s.c. eliminated the inhibition of IL1β in *trpv1ko* endotoxemic mice at 6 h. (n = 5/group) Unpaired two-sided Student's *t*-test.

Data are presented as the mean ± SEM. $^{**}P < 0.01$, $^{*}P < 0.05$; ns, not significant.

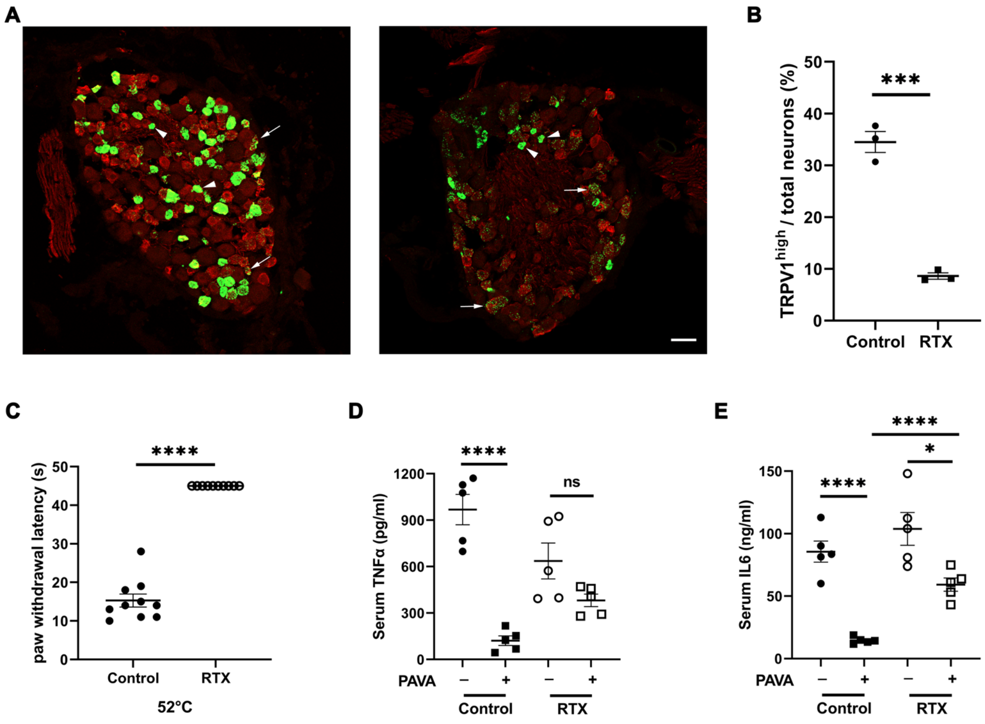

Figure S2. TRPV1+ neuron ablation suppressed PAVA-induced anti-inflammatory effects.

(A, B) Immunostaining images (A) and quantification (B) of TRPV1+ neurons showed that RTX treatment ablated TRPV1+ neurons in C6-C8 DRGs. Arrowheads indicate high-level TRPV1 expression and arrows indicate low-level TRPV1 expression. (n = 3/group) Scale bar: 50 μm.

(C) Vehicle-treated and RTX-treated mice were assayed in the hot plate test (52°C) for latency to hindpaw lifting/licking/flinching. (n = 10/group)

(D, E) RTX pretreatment significantly suppressed the anti-inflammatory effects of PAVA treatment on TNFα (D) and IL6 (E) expression. (n = 5/group)

Data are presented as the mean ± SEM. Unpaired two-sided Student's *t*-test. ****$P < 0.0001$, ***$P < 0.001$, **$P < 0.01$, *$P < 0.05$; ns, not significant.

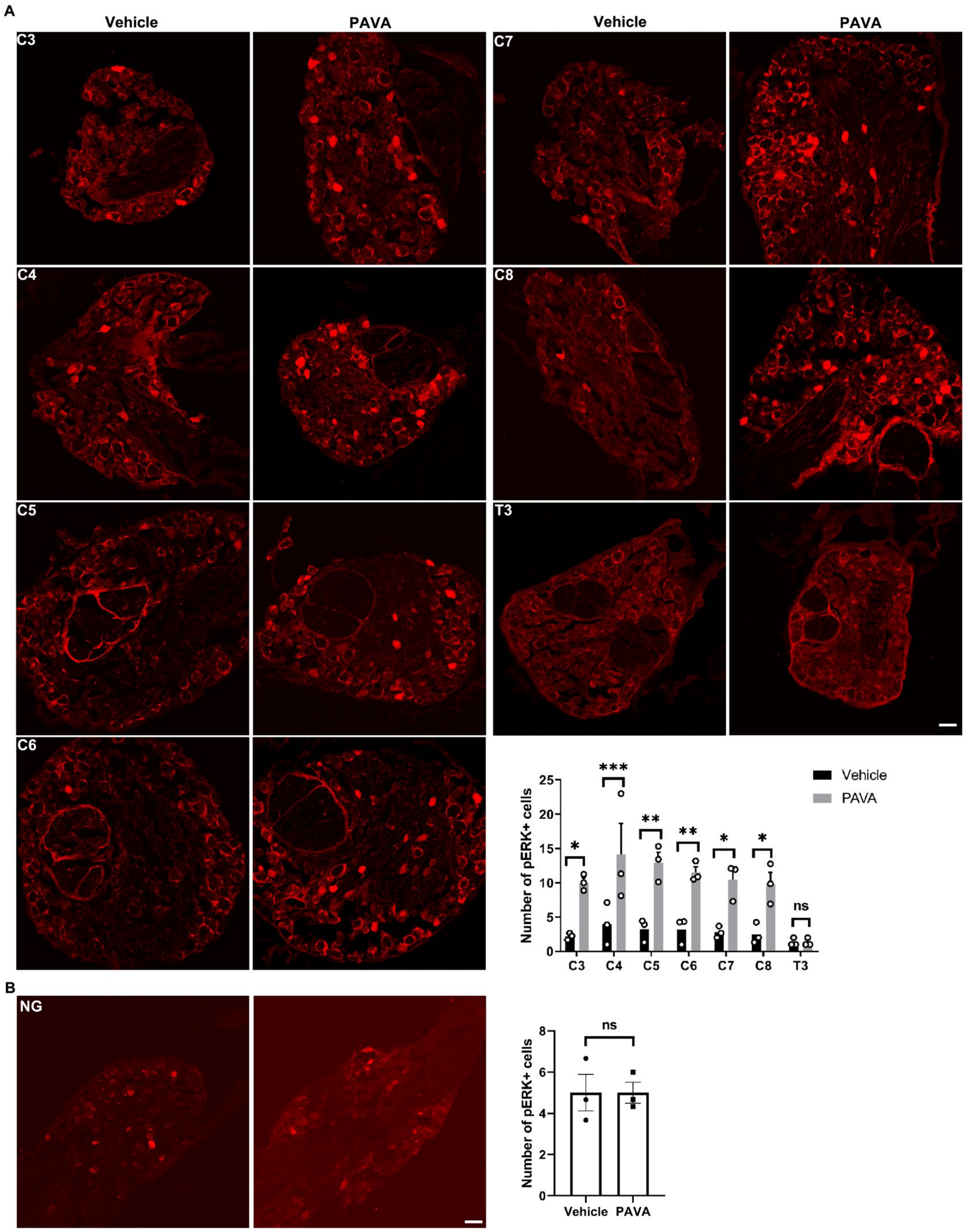

Figure S3. PAVA nape s.c. induced pERK expression in cervical DRGs rather than NGs.

(A) Representative sections in C3-C8 and T3 DRGs showed pERK expression following PAVA administration. The counts of pERK+ cells in C3-C8 and T3 DRGs. (n = 3/group)

(B) Representative sections showed pERK expression in NGs following PAVA administration. Quantification of pERK+ cells in NGs. (n = 3/group)

Data are presented as the mean ± SEM. Two-way ANOVA with Bonferroni post tests (A), unpaired two-sided Student's *t*-test (B). ***$P < 0.001$, **$P < 0.01$, *$P < 0.05$; ns, not significant. Scale bars: 50 μm.

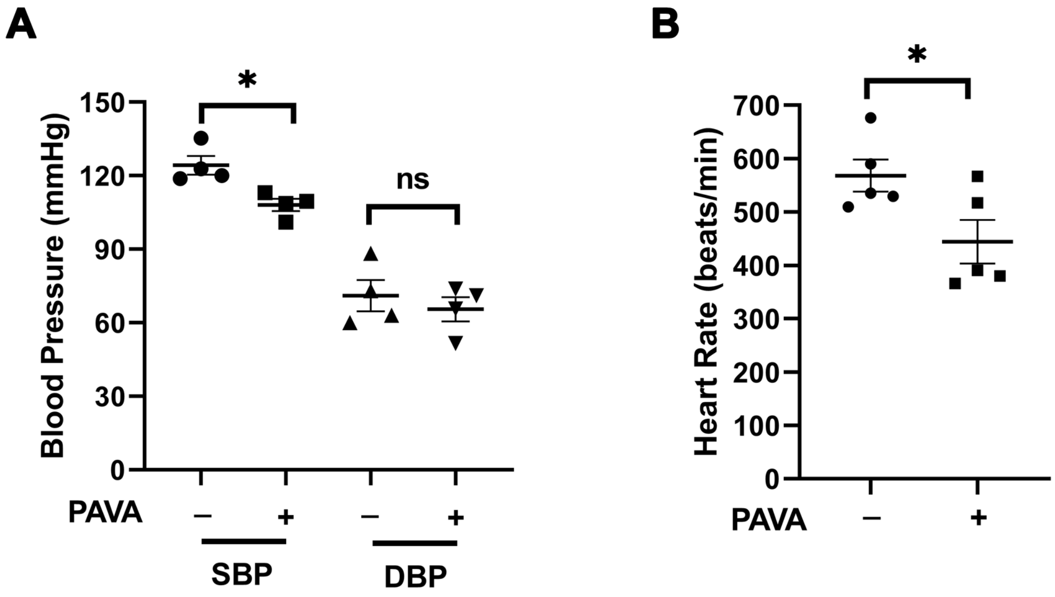

Figure S4. PAVA treatment affected blood pressure and heart rate of the mice.

(A) PAVA nape s.c. downregulated systolic blood pressure (SBP) but not diastolic blood pressure (DBP). (n = 4/group)

(B) PAVA treatment elicited the decrease in the heart rate. (n = 5/group)

Data are presented as the mean ± SEM. Unpaired two-sided Student's *t*-test. $^*P < 0.05$; ns, not significant.

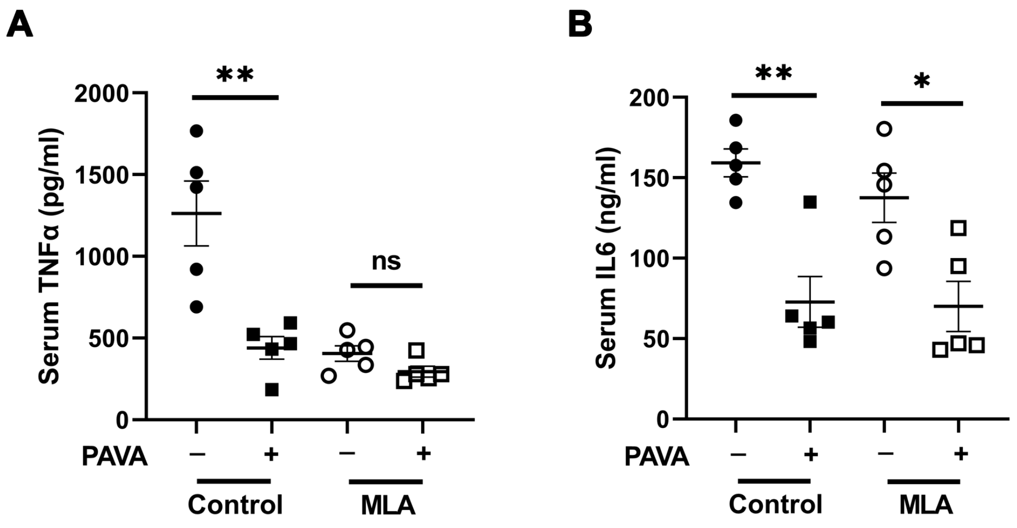

Figure S5. Blocking α7nAChR is not enough to abolish the anti-inflammatory effects of PAVA treatment.

(A) Methyllycaconitine citrate (MLA), a selective α7nAChR antagonist, inhibited LPS-induced TNFα production.

(B) MLA did not affect the anti-inflammatory effects of PAVA treatment on IL6 expression.

Data are presented as the mean ± SEM. Unpaired two-sided Student's *t*-test, n = 5/group. $^{**}P < 0.01$, $^{*}P < 0.05$; ns, not significant.
